# Magnetic resonance imaging of the brachial plexus. Part 1: Anatomical considerations, magnetic resonance techniques, and non-traumatic lesions

**DOI:** 10.1016/j.ejro.2021.100392

**Published:** 2021-12-20

**Authors:** Pawel Szaro, Alexandra McGrath, Bogdan Ciszek, Mats Geijer

**Affiliations:** a Department of Radiology, Institute of Clinical Sciences, Sahlgrenska Academy, University of Gothenburg, Gothenburg, Sweden; b Department of Musculoskeletal Radiology, Sahlgrenska University Hospital, Gothenburg, Sweden; c Umeå University, Faculty of Medicine, Department of Clinical Sciences, Professional Development. Umeå University, Faculty of Medicine, Department of Surgical and Perioperative Sciences, Sweden; d Department of Descriptive and Clinical Anatomy, Centre of Biostructure Research, Medical University of Warsaw, Chałubinskiego 5, 02-004 Warsaw, Poland; e Department of Neurosurgery, Bogdanowicz Memorial Hospital, Niekłanska 4/24, 03-924 Warsaw, Poland; f Department of Clinical Sciences, Lund University, Lund, Sweden

**Keywords:** BP, brachial plexus, DTI, diffusion tensor imaging, MRP, multiplanar reformation, OBPP, obstetric brachial plexus palsy, STIR, short tau inversion recovery, TI, inversion time, TOS, thoracic outlet syndrom, TSE, turbo spin-echo, Brachial plexus, Injury, Tumor, Compression, Neuropathy

## Abstract

For magnetic resonance imaging (MRI) of non-traumatic brachial plexus (BP) lesions, sequences with contrast injection should be considered in the differentiation between tumors, infection, postoperative conditions, and post-radiation changes. The most common non-traumatic inflammatory BP neuropathy is radiation neuropathy. T2-weighted images may help to distinguish neoplastic infiltration showing a high signal from radiation-induced neuropathy with fibrosis presenting a low signal.

MRI findings in inflammatory BP neuropathy are usually absent or discrete. Diffuse edema of the BP localized mainly in the supraclavicular part of BP, with side-to-side differences, and shoulder muscle denervation may be found on MRI.

BP infection is caused by direct infiltration from septic arthritis of the shoulder joint, spondylodiscitis, or lung empyema.

MRI may help to narrow down the list of differential diagnoses of tumors. The most common tumor of BP is metastasis. The most common primary tumor of BP is neurofibroma, which is visible as fusiform thickening of a nerve. In its solitary state, it may be challenging to differentiate from a schwannoma.

The most common MRI finding is a neurogenic variant of thoracic outlet syndrome with an asymmetry of signal and thickness of the BP with edema. In abduction, a loss of fat directly related to the BP may be seen.

Diffusion tensor imaging is a promising novel MRI sequences; however, the small diameter of the nerves contributing to the BP and susceptibility to artifacts may be challenging in obtaining sufficiently high-quality images.

## Highlights

• MRI allows narrowing the list of differential diagnoses of brachial plexus lesions.
• MRI helps to distinguish neoplastic infiltration from radiation neuropathy in T2-weighted images.
• Differentiation between tumors, infection, postoperative conditions and post-radiation changes is possible with contrast.
• MRI helps to determine the extent of the infection.
• Diffusion tensor MRI is a promising method for brachial plexus assessment.

## 1 Introduction

The current article is focused on diagnostic imaging with magnetic resonance (MRI) and its role in diagnosing common BP non-traumatic lesions from both radiologists' and surgeons' perspectives. Traumatic lesions are covered in Part 2 (Magnetic resonance imaging of the brachial plexus. Part 2: Traumatic injuries).

### 1.1 Anatomical variations of the brachial plexus

In 75% of cases, the BP is composed of the anterior branches of the spinal nerves C5—Th1 [1]. There are two common types of anatomical variations in the supraclavicular part of the BP. The first group includes pre- or postfixed BP. The second group includes a variety of variants of communication between different parts of the BP.

Pre- or postfixed BP is a term concerning the BP position in relation to the craniocaudal axis of the spinal cord. The contribution of fibers from C4 is called prefixed, while the contribution from Th2 plexus is called postfixed. Significant discrepancies can be observed in determining the frequency of these variants from 15% [2] to 63% [3] for the prefixed and 0.66% [4] to about 58% [5] for the postfixed BP. The results of the cited works differ significantly due to the size of the patient cohorts. In an extensive study of 200 BP, the prefixed variant incidence was determined at 25.5% and the postfixed at 2.5% [1]. Variations of the divisions, the fascicles, and the BP branches are expected and estimated at 50%, while root and trunk variants are rare [1]. Some nerves show a relatively high variation rate, like the long thoracic nerve or medial antebrachial cutaneous nerve [1], [6], but these nerves are less critical in diagnosing plexus injury.

### 1.2 Radiological anatomy of the brachial plexus

Anatomical assessment of the BP starts with evaluating the spine, spinal cord, and roots of the spinal nerves. It should be noted that the roots of the spinal nerves are located in the intradural space in the subarachnoid space of the vertebral canal (Fig. 3). The ventral and dorsal roots of the spinal nerve unite in the intervertebral foramen (anatomically called the trunk of the spinal nerve, which is not the same as the trunk of the BP). The sensory ganglion, located on the dorsal root (dorsal root ganglion), is a critical MRI landmark for avulsion injuries (Fig. 3 and 4). After leaving the intervertebral foramen, the spinal nerve divides into ventral and dorsal branches. The extensions of the ventral branches of the spinal nerves C5–Th1 are called the roots of the BP. Identifying the root of the BP should start between the transverse processes and space between the scalenus anterior and the scalenus posterior muscle. The names of the BP roots come from the spinal cords' segments, e.g., root C5 of the BP (Fig. 4).

Furthermore, the C5 and C6 roots fuse to form the upper trunk, the C7 root extends as the middle trunk, and the C8 and Th1 roots unite to form the lower trunk. Each trunk is divided into anterior and posterior divisions, which form the cords of the BP when joined. The medial cord is formed as a prolongation of the lower trunk. The lateral cord is formed by anterior divisions of the middle and upper trunk (Fig. 4, Fig. 5, Fig. 6, Fig. 7). All posterior divisions form the posterior cord. The cords contain axons originating from different spinal cord segments [1], [4], [6], [7] (Fig. 5, Fig. 6).

### 1.3 Anatomical advice for BP identification on MRI

Identifying BP components might be difficult, but helpful tricks can make the process easier and more effective. The tortuosity of BP increases from C5 to T1 [8] thus, MPR reconstructions helps in identifying.

#### 1.3.1 The roots and trunks of the BP

The first step is determining whether the spinal nerves C5 - Th1 form the BP (Fig. 3). The spinal nerves' anterior and posterior roots can be found in the spinal canal and are followed to ensure continuity with the spinal cord. This way, root avulsion can be ruled out. Since the spinal nerves' roots and the BP roots run in the coronal plane, they are easy to find on transverse and coronal sections [9], [10], [11]. By following the roots of the BP, identifying the trunks usually becomes easier (Fig. 4). Identification of the Th1 root may cause some problems because it is significantly smaller than the other roots, but by performing MPR, a small nerve passing superiorly and laterally to the column of the first rib can be seen (Figs. 4 and 5).

#### 1.3.2 The cords and their divisions

At the level of the scalenus muscles, the trunks of the BP descend inferolateral and slightly anteriorly and divide into anterior and posterior divisions. All trunks run parallel and then converge and become closer (Fig. 5, Fig. 6, Fig. 7). The posterior divisions from all trunks unite to form the posterior cord. The anterior division from the lower trunk continues as the medial cord, while the anterior divisions from the upper and middle trunk form the lateral cord. The cords' names originate from the axillary artery; thus, it is easiest to identify the cords on the sagittal cross-sections. As the BP runs irregularly obliquely in the axillary cavity, it can be hard to identify and assess all components in one cross-section. Hence, it is worth using all three planes or MPR (Fig. 5, Fig. 6, Fig. 7). In the coronal plane (Fig. 7a), the medial cord is located most inferiorly, and the lateral cord most superiorly. The posterior cord is usually located between the medial and lateral cord in the sagittal plane (Fig. 7b).

#### 1.3.3 The most prominent branches of the brachial plexus

The radial nerve is the largest and the most posteriorly located nerve running toward the humerus (Fig. 7). The axillary nerve is the most superior of the laterally located branches and runs somewhat horizontally and obliquely towards the surgical neck of the humerus (Fig. 7). The median and ulnar nerves can be harder to distinguish because they run very close together on the anteromedial circumference of the axillary artery (Fig. 7).

### 1.4 BP imaging strategies

**Fig. 1:**
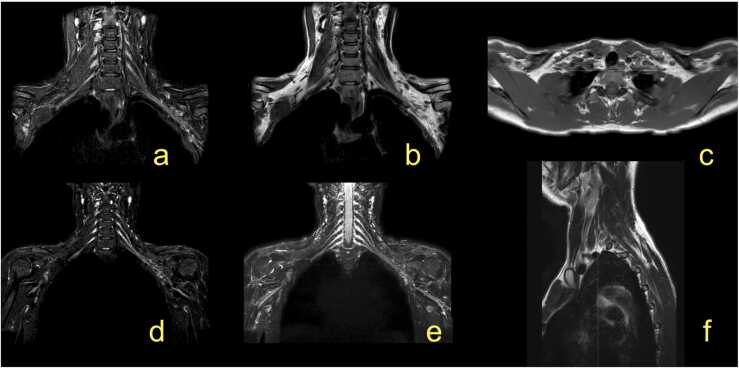
The magnetic resonance imaging protocol used at the authors' institution. (a) T2-weighted modified DIXON sequence with fat suppression, (b) T2-weighted modified DIXON sequence without fat suppression, (c) T1-weighted turbo spin-echo (TSE) sequence, (d) 3D neurography, (e) neurography MIP 3 mm, (f) T2-weighted TSE sequence.

**Fig. 2:**
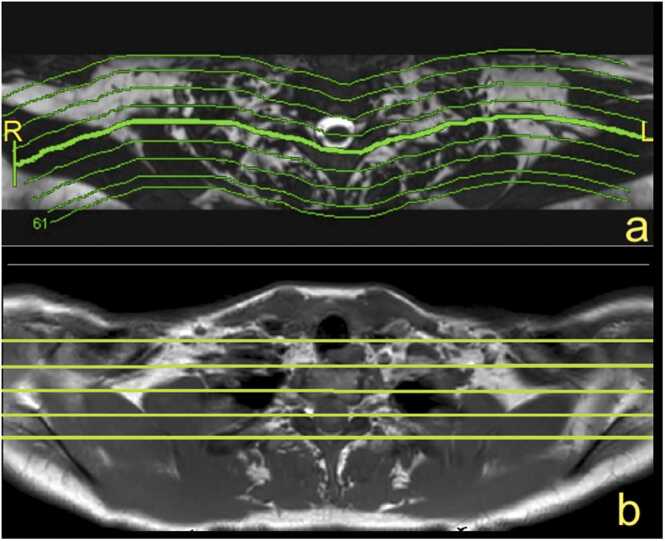
(a) The paracoronal plane follows the direction of nerves and brachial plexus. (b) The anatomical coronal plane is used at the authors' institution.

**Fig. 3:**
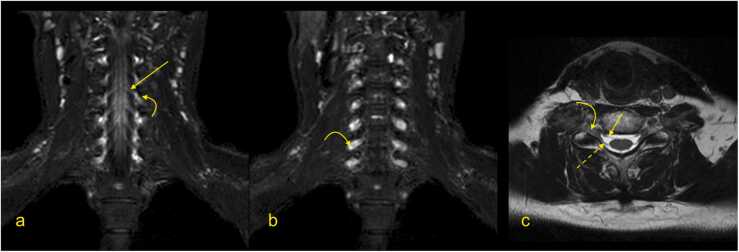
Magnetic resonance imaging of the brachial plexus. (a and b) T2-weighted DIXON sequence with fat suppression, (c) T2-weighted turbo spin-echo sequence). Curved arrow – the ganglion of the spinal nerve, arrow – the anterior root of the spinal nerve, dashed arrow – the posterior root of the spinal nerve.

**Fig. 4:**
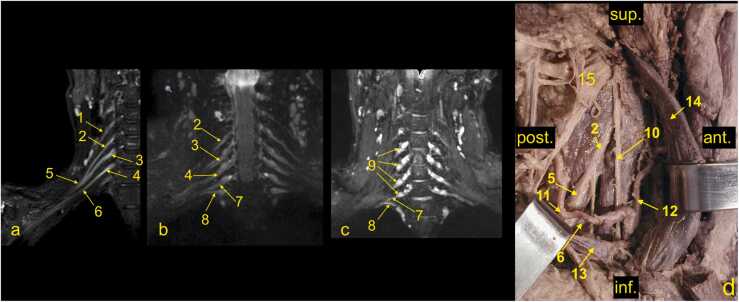
(a-c) The roots and trunks of the normal brachial plexus (BP) on magnetic resonance imaging. (d) cadaver specimen, anterolateral view. 1 – the C4 spinal nerve (not contributing to the BP), 2 – the C5 root, 3 – the C6 root, 3a- the anterior division of the middle trunk, 4 – the C7 root, 5 – the upper trunk, 6 – the middle trunk, 7 – the C8 root, 8 – the Th1 root, 9 – the ganglia of the spinal nerves C5-C8, 10 – the phrenic nerve, 11 – the suprascapular artery, 12 – the ascending cervical artery, 13 – the subclavian vein, 14 – the internal jugular vein, and 15 – the nerve point of the neck. Ant.; anterior, post.; posterior, sup.; superior, inf.; inferior.

**Fig. 5:**
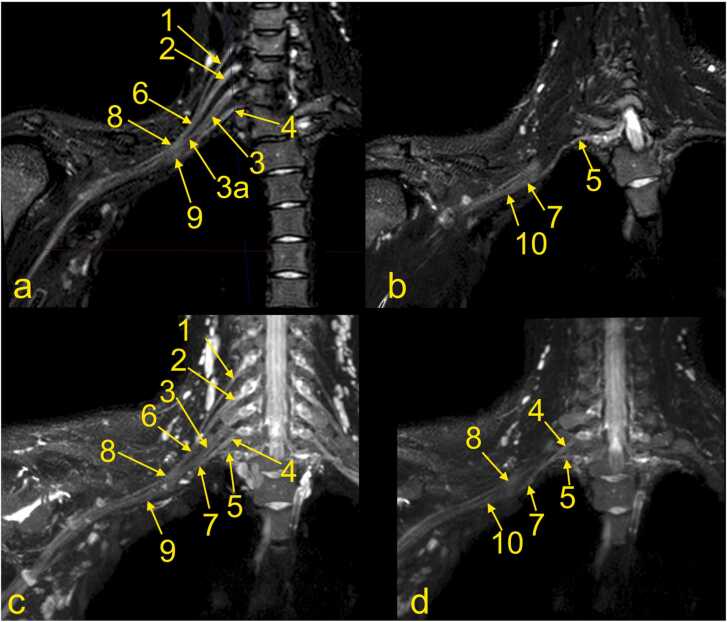
MRI of the brachial plexus, right side. (a and b) T2-weighted DIXON sequence with fat suppression. (b) MPR of the C8 root. (c and d) Neurography. 1 – the C5 root, 2 – the C6 root, 3 – the middle trunk, 4 – the C8 root, 5 – the Th1 root, 6 – the upper trunk, 7 – the lower trunk, 8 – the lateral cord, 9 – the medial cord, and 10 – the posterior cord.

In the adult, a proper anatomical approach is crucial for imaging of the BP disorders, and several MRI protocols have been proposed [9]. The BP does not run in a single anatomical plane (Fig. 1, Fig. 2, Fig. 3, Fig. 4, Fig. 5), which requires imaging in irregular planes. Isovolumetric 3D sequences enabling reconstruction in different planes or multiplanar reformation (MPR) are essential techniques, especially in newborns [12]. MR neurography allows high-quality visualization of the BP and its branches [9], [11], [13], [14]. It may be used in routine protocols in combination with other sequences (Fig. 1). The MRI protocol for injury of the brachial plexus is presented in the Table 1. Additionally, MRI of the cervical spine is performed to exclude injury to the roots of the spinal nerves.

**Table 1:** MR imaging of the adult brachial plexus after trauma.

Sequence	TE (ms)	TR (ms)	FOV (mm) RL FH AP	Voxel (mm)	Gap (mm)
T2-weighted (in-phase and out of phase DIXON) Coronal section	90	3000	360 320 110	0,7 × 0,8 × 3,5	1
T2-weighted sagittal section	90	4300	240 300 240	0,7 × 0,8 × 3,5	1
T1-weighted TSE	18	633	360 353 160	0,9 × 1,1 × 3,5	0,35
Nerve view	170	220	390 300 170	1,2 × 1,2 × 2	-1

Abbreviations: AP – the anterior-posterior dimension, FH- feet- head dimension, FOV – the field-of-view, RL- the right-left dimension, TE – the echo time, TR - the repetition time, TSE – the turbo spin echo.

It is still unclear which method is more reliable in assessing a complex injury or pathology of the BP. The intradural part of the spinal nerves can be evaluated by CT myelography and MR myelography [9], [15], [16]. Sequences with intravenous (i.v.) gadolinium injection are often used when tumor or infection is suspected, in postoperative cases, and for post-radiation injuries [7], [9], [14]. The entire BP examination often requires the use of an 8- or 16-channel neurovascular coil. Fast sequences allow for a shorter examination time with fewer motion artifacts [9], [17]. MR neurography can be obtained with 1.5 T and 3 T MR scanners; however, the optimal field strength is unclear. To receive a high signal from the BP and a low signal from fat, TI = 180 ms is recommended at 1.5 T for obtaining MR neurography. The delineation of the BP and its branches is better at 3 T than 1.5 T [7], [9], [18]. The routine protocol should include T1-weighted and T2- weighted sequences [9]. The supraclavicular part can be easily assessed on coronal or paracoronal images and the infraclavicular part on axial images, but the best assessment can be achieved using 3D sequences (Fig. 1, Fig. 2). For the anatomy of the newborn, see the section on obstetric BP palsy.

### 1.5 Non-traumatic neuropathy of the brachial plexus

This group includes primary or secondary malignant or benign tumors, infections, inflammatory neuritis or neuropathy, compression of the BP, and thoracic outlet syndrome (TOS) [9], [10], [11], [19]. About three-quarters of cases belonging to this group are related to radiation neuropathy, infiltration, or compression by breast or lung cancer [20].

#### 1.5.1 Radiation neuropathy

Radiation neuropathy is the most common non-traumatic inflammatory cause of BP neuropathy and is seen when the cumulative dose of radiation exceeds 60 Gy [20]. It may manifest itself as pain and weakness of the arm flexors and shoulder abductors, and paresthesia. It occurs earlier than six months after radiotherapy, it is classified as acute and probably ischemic in origin, while it is classified as chronic and related to fibrosis after six months. The upper trunk is most often affected. It can be hard to distinguish between radiation-induced changes and direct infiltration by tumor or other pathology related to the intervertebral disk clinically, and magnetic resonance imaging (MRI) is often recommended. However, positron emission tomography (PET)/CT is appropriate for differentiating radiation ischemia from radiation fibrosis. In unclear cases, a biopsy may be indicated [19], [21]. T2-weighted images may help distinguish neoplastic infiltration, which exhibits a high signal from radiation-induced neuropathy fibrosis showing low signal (Fig. 8, Fig. 9). Very rarely, fibrosis may also show a somewhat higher signal, probably due to the overlap with other conditions and changes related to tumor treatment. The administration of i.v. contrast is usually not conclusive since tumor infiltration, lesions related to treatment, and fibrosis may show a similar enhancement [20].

**Fig. 6:**
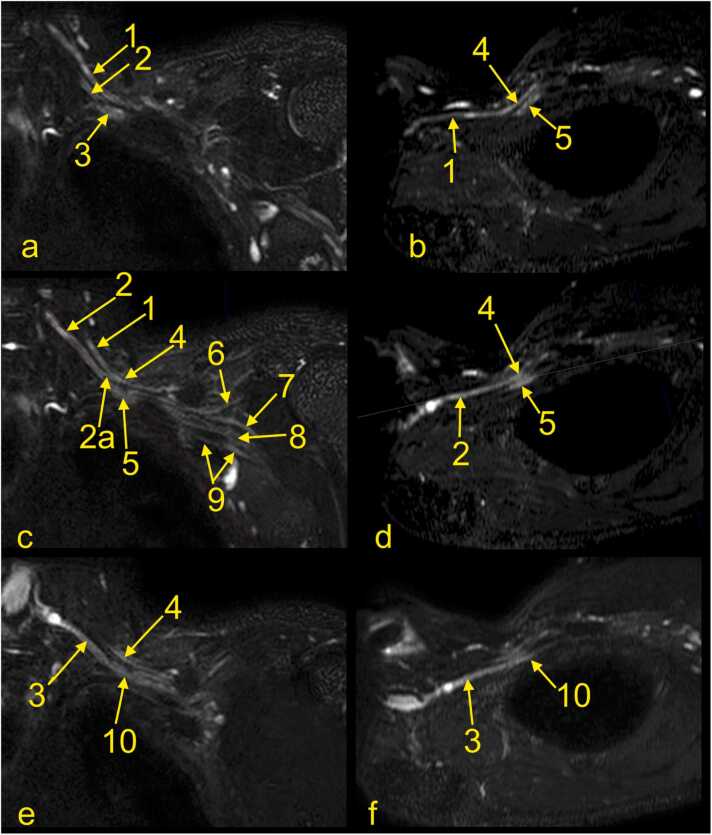
The cords of the left brachial plexus. (a, c, e) Coronal sections, (b) oblique section through the upper trunk, (d) oblique section through the middle trunk, and (f) oblique section through the inferior trunk. 1 – the upper trunk, 2 – the middle trunk, 2a – the anterior division of the middle trunk, 3 – the lower trunk, 4 – the lateral cord, 5 – the posterior cord, 6 – the axillary nerve, 7 – the radial nerve, 8 – the median nerve, 9 – the ulnar nerve, and 10 – the medial cord.

**Fig. 7:**
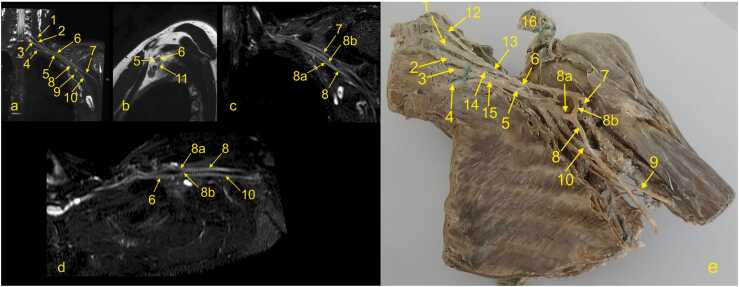
The cords and nerves of the left brachial plexus. (a-d) Magnetic resonance imaging of the left brachial plexus (MRI-neurography) with anatomical correlation. (e) A plastinated specimen of the left brachial plexus, anterior view, where the left hemithorax and cervical spine are dissected. 1 – the C6 root, 2 – the C7 root, 3 – the C8 root, 4 – the Th1 root, 5 – the medial cord, 6 – the lateral cord, 7 – the musculocutaneous nerve, 8 – the median nerve, 8a – the medial root, 8b – the lateral root, 9 – the ulnar nerve, 10 – the radial nerve, 11 – the posterior cord, 12 – the C5 root, 13 – the upper trunk, 14 – the middle trunk, 15 – the lower trunk, and 16 – the clavicula.

**Fig. 8:**
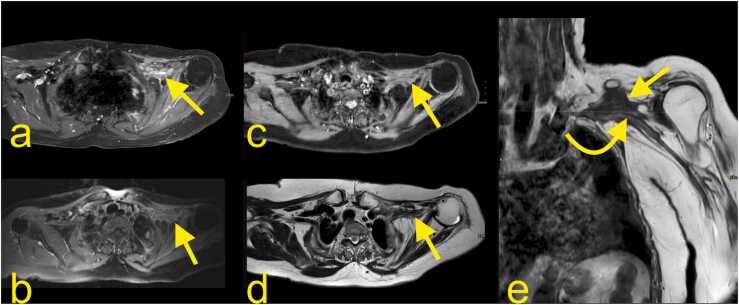
After radiation treatment, an 80-year-old patient with regional recurrence of previous breast cancer in the left axillary region showed promising results. However, increasing pain in the left arm, forearm, and hand leads to clinical suspicion of recurrence. (a) Initial MRI with TSE Spectral Attenuated Inversion Recovery (SPAIR) T1-weighted imaging with iv contrast before radiation showed an irregular mass in the left axillary cavity involving the BP (curved arrow) and axillary vessels (straight arrow). (b) The pain decreased, and a good effect of radiation was seen after six months on the T1-weighted TSE SPAIR MRI with i.v. contrast. One year later, the patient presented with increasing diffuse pain. The new MRI revealed radiation fibrosis (straight arrow) without the recurrence of the metastasis.

**Fig. 9:**
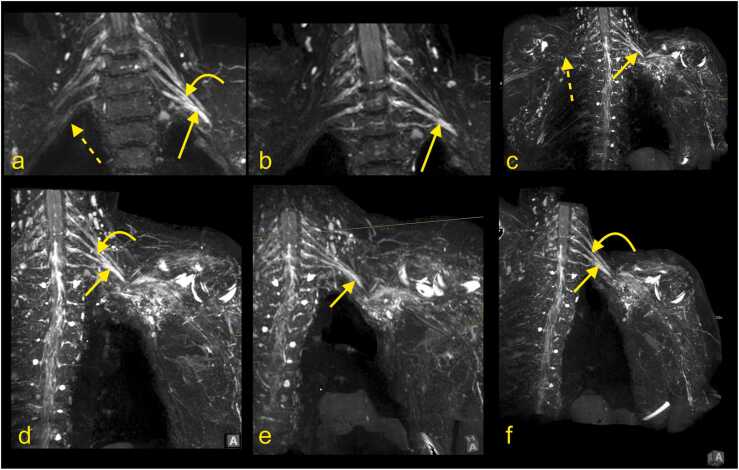
Continuation of the patient from the previous figure. MRI neurography (a-e) with 3D- Maximum Intensity Projection (MIP) reconstructions (d-e) showed the discreet asymmetry of the BP; the left one is more prominent (straight arrows show the lower trunk, the curved arrows show the middle trunk, the straight dashed arrows show the normal BP). The normal signal of the BP is not visible on the level of the radiation fibrosis. However, no discontinuity was demonstrated.

#### 1.5.2 Inflammatory brachial plexus neuropathy

Inflammatory BP neuropathy manifests as an acute onset of burning pain in the shoulder, subsequent sensory disturbances, muscle weakness, and atrophy [19], [22], [23]. The upper part of the plexus is usually affected. The differential diagnosis includes radiculitis, spondylodiscitis, and rotator cuff rupture. The etiology is unclear, and a form of autoimmunological disorder or viral infection has been advanced as possible causes [24], [25]. MRI findings are usually absent; if present, they are discrete or unspecific. A difference between the affected and unaffected sides can be shown. Diffuse edema of the BP localized mainly in the upper part, and shoulder muscle denervation may be found by MRI [22]. The muscle denervation is predominantly seen in the muscles supplied by the suprascapular nerve (Parsonage-Turner syndrome), the axillary nerve, or the long thoracic nerve [19].

**Fig. 10:**
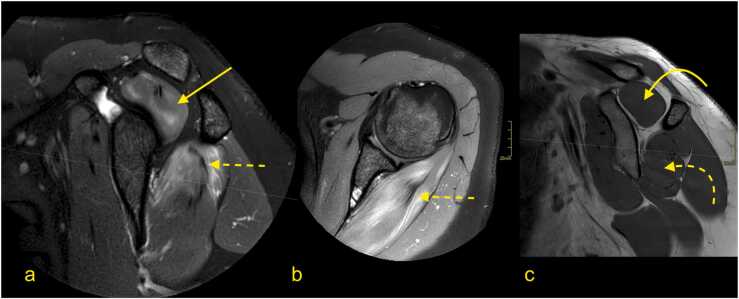
Parsonage-Turner syndrome. A 31-year-old patient with pain in the left shoulder. (a) T2-weighted TSE MRI with fat suppression, (b) PD-weighted MRI with fat saturation, and (c) T1-weighted TSE MRI. There was diffuse muscle edema in the supraspinatus (arrow) and infraspinatus (dashed arrow) muscles without atrophy (curved arrow and curved dashed arrow). This represents the involvement of the suprascapular nerve; however, no lesion was demonstrated in the infraclavicular part of the BP.

Parsonage-Turner syndrome is the classic presentation of a BP inflammatory neuropathy and starts with a unilateral onset of continuous pain in the shoulder and arm. As it causes muscle denervation, it may be confused with rotator cuff pathology. Usually, the diagnosis is based on medical history, clinical examination, and electromyography. MRI is usually applied in atypical cases or if there is an unclear history of trauma. MRI may reveal nerve edema and higher signals on T2-weighted sequences (Fig. 10) [26], [27].

Chronic inflammatory demyelinating polyneuropathy (CIDP) is a rare condition with inflammation of axons because of myelin destruction. MRI of the nerves usually reveals thickening of the nerves and contrast enhancement [24].

#### 1.5.3 Infection

Fever, quick weakness, and painful swelling of the arm may indicate BP infection. Infection of the BP is rare and usually spreads from structures located close to the BP, i.e., respiratory tract infection. After BP surgery, complications might even cause septic myositis, septic arthritis of the shoulder, spondylodiscitis, or lung empyema [28]. In the acute phase, fatty infiltration and swollen nerves can be seen as the higher signal on T2-weighted images and a linear increased signal on T1-weighted images, which indicates lesion. The higher signal on neurography can persist even in the chronic phase. After i.v. contrast administration variable enhancement pattern of BP and in the adjacent structures is noticed depending on infection extension [28].

MRI helps to determine the extension of the infection and allows to assess whether there is an abscess. Relation to axillary vessels and adjacent structures may be evaluated, essential before puncture or drainage. The etiology may be bacterial [28], [29] or viral [25]. A broad spectrum of etiologies requires material aspiration to identify the pathogens to guide antibiotic therapy [19].

#### 1.5.4 Tumors

##### 1.5.4.1 Metastases

**Fig. 11:**
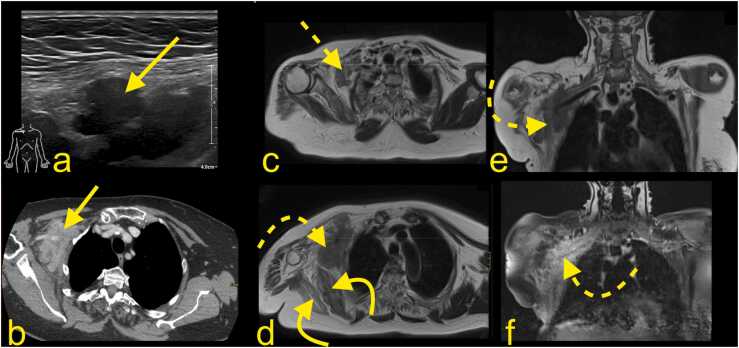
A 78-year-old patient underwent a mastectomy and partial axillary evacuation 15 years ago. Now, 1–2 months ago, she reported a swollen arm after trauma. The radiography showed no fracture. Ultrasound (a) revealed a mass in the right axillary cavity (arrow on a-e). (b) No thrombus in the axillary vessels or metastasis in the lungs were revealed on thoracic CT with i.v. contrast. The mass in the right axillary cavity (straight arrow on a and b) was biopsied, and breast cancer metastasis was confirmed. Radiation was administered with good clinical response (dashed arrow on c). After two years without symptoms, gradual clinical progress, and increasing weakness in the right arm, there was a suspicion of metastasis progress (d, e and f). Repeated MRI of the BP with (d) T2-weighted axial imaging, (e) T1-weighted TSE, and (f) T1-weighted imaging with i.v. contrast and fat suppression showed the metastasis (curved dashed arrow) with about 20% volume progress and infiltration of the right BP (dashed arrow). Muscle atrophy is seen on the right side (curved arrow).

**Fig. 12:**
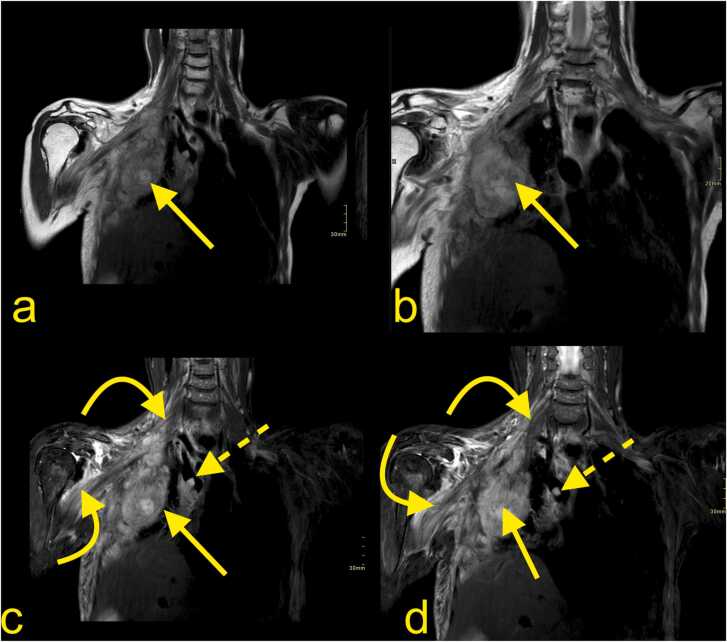
A 62-year-old patient with generalized HER2-positive breast cancer since 2001. There were manifestations in the lungs, lymph nodes, and skeleton (straight arrow). The patient's biggest problem was swelling, aches, poor motor function, and sensation around the right scapula, in the shoulder, and arm. MRI of the BP with (a, b) T2-weighted DIXON TSE and (c, d) T2-weighted DIXON TSE with fat suppression showed an extensive tumor mass (straight arrow) originating from the thoracic wall and infiltration of the BP. Both the supra- and infraclavicular parts of the BP are swollen with increased signals (curved arrow). Vascular invasion is seen in the superior vena cava (straight dashed arrow).

Metastatic lesions are more common than primary tumors of the BP. Metastasis may purely compress the BP (Fig. 11) or infiltrate it (Fig. 12). The BP area is a common site for metastases from breast cancer, lung cancer, and lymphoma (neurolymphomatosis). Other relatively frequent origins of metastases are head and neck tumors [19]. Most metastases show the same pattern of low signal on T1-weighted and higher signal on T2-weighted sequences (Fig. 11, Fig. 12), but desmoplastic breast cancer may occasionally show a lower signal on T2-weighted sequences [19], [20]. Radiation-induced sarcoma of the BP is uncommon [10], [19]. A Pancoast tumor (primary lung cancer) may infiltrate the C8 and Th1 root of the BP or the lower trunk. Because the stellate and inferior cervical ganglion is directly related to the lung apex, it can be infiltrated; thus, Horner syndrome may be seen in about 20% of cases [20].

##### 1.5.4.2 Primary nerve tumors

**Fig. 13:**
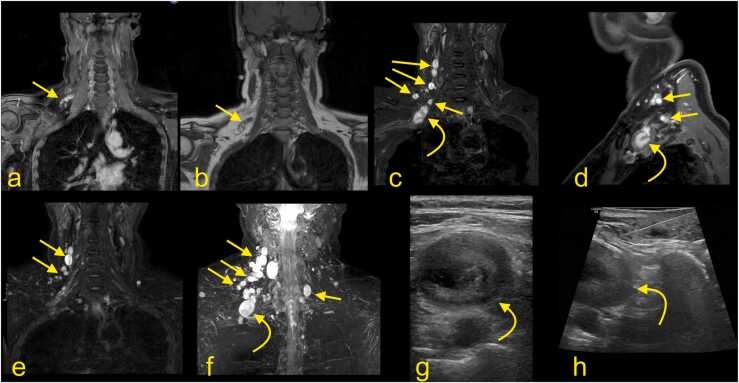
A 58-year-old patient presented with radiating pain to the right arm about eight years ago. MRI of the BP with (a) T1-weighted imaging with i.v. contrast and (b) T1-weighted TSE imaging showed small BP tumors on the right side. The patient had been operated on ten years earlier in the same region; the histopathological diagnosis was plexiform neurofibroma. Now, the patient was complaining of increasing symptoms from the left side of the neck. A hard mass could be palpated in the lateral neck triangle, and the patient's arm was tired after use. The new MRI of the BP with (a) T1-weighted imaging with fat suppression and i.v. contrast, (b) T1-weighted imaging, (c) T2-weighted mDIXON imaging, (d) T1-weighted mDIXON TSE with i.v. contrast, (e) T2-weighted mDIXON TSE imaging, and (f) T2-weighted mDIXON TSE MIP reconstruction showed multiple solid tumors in the lateral triangle of the neck (straight arrows). Because of the unclear symptoms and enlarged lymph nodes, the multidisciplinary team requested a biopsy. An ultrasound-guided core biopsy (g, h) with a 16-gauge needle (dashed arrow) from the most prominent lesion (curved arrow) was performed; no malignant cells were present.

**Fig. 14:**
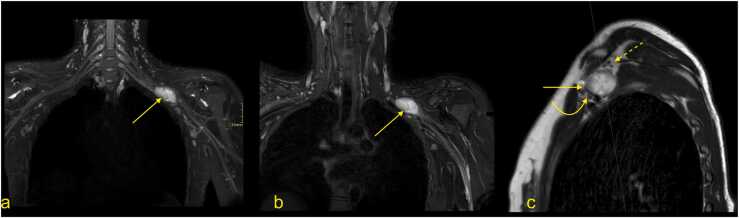
An 18-year-old patient presented with numbness pain in the left arm, forearm, and fingers 2–4. The patient's mother has a history of neurofibromatosis. MRI with (a, b) neurography and (c) T2-weighted TSE imaging showed a well-defined tumor within the medial cord of the BP, size 2 × 4×2 cm (straight arrow). The tumor displaced the lateral cord anteriorly (curved arrow) and the posterior cord superiorly (dashed arrow). The lesion was resected, and the histopathological diagnosis was neurilemmoma (Schwannoma).

Primary nerve tumors are rare, the most common being neurofibroma (Fig. 13) [30]. About 30% are seen in patients with neurofibromatosis type 1, often with multiple tumors. A solitary variant may be difficult to differentiate from a schwannoma (Fig. 14). A low signal on T2-weighted imaging may be seen in the central part of the neurofibroma, called the target sign (Fig. 13) [31]. Both schwannoma and the focal form of neurofibroma appear as a well-defined mass along the nerve's long axis, enhancing avidly [32]. In rare cases of neurofibromatosis, a malignant nerve sheath tumor may develop, which forms a progressively growing irregular, ill-defined heterogeneous mass, infiltrating the neighboring structures. However, MRI shows relatively low sensitivity (43–60%) [19]. PET-CT shows over 90% specificity and sensitivity in differentiating between neurofibroma and malignant nerve sheath tumors [33], [34].

Another rare tumor is the plexiform neurofibroma [19], [20], [32], which belongs to a group of rare benign non-neural sheath lesions originating from the Schwann or perineurial cells called localized hypertrophic neuropathy of the peripheral nerve as lipomas, vascular tumors, or cysts [35].

Tumor or tumor-like lesions close to the BP may compress or displace it (Fig. 11, Fig. 12). A typical example is a lipoma growing in the axial cavity. Other benign lesions that may compress the BP are desmoid tumor, lymphangioma, or hematoma. Direct MRI diagnosis is impossible in most benign soft tissue lesions except lipoma; thus, a biopsy may be considered [36].

##### 1.5.4.3 Thoracic outlet syndrome

**Fig. 15:**
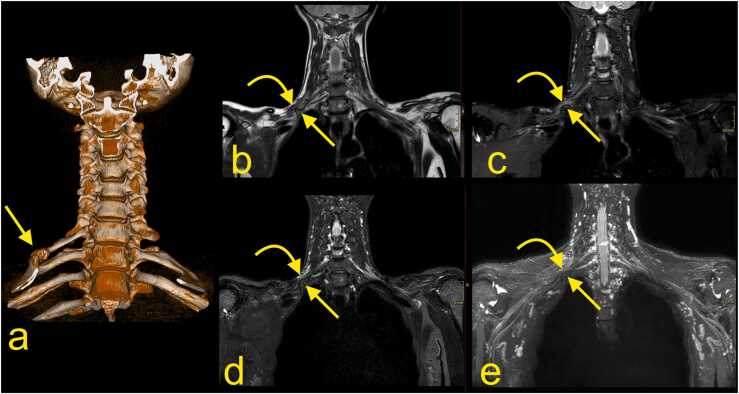
A 42-year-old patient with an accessory cervical rib on the right side shown in (a) CT with 3D reconstruction (straight arrow) was detected one year earlier because of pain on the right side of the neck when lying down and during specific movements. The patient now presented with weakness of the right-hand muscles and occasional paresthesia in the right arm. Clinically, thoracic outlet syndrome was suspected. MRI with (b) T2-weighted DIXON TSE, (c, d) T2-weighted DIXON TSE with fat suppression, and (e) MR neurography showed that the accessory rib (straight arrow) dislocated and distorted the lower trunk of the BP (curved arrow).

**Fig. 16:**
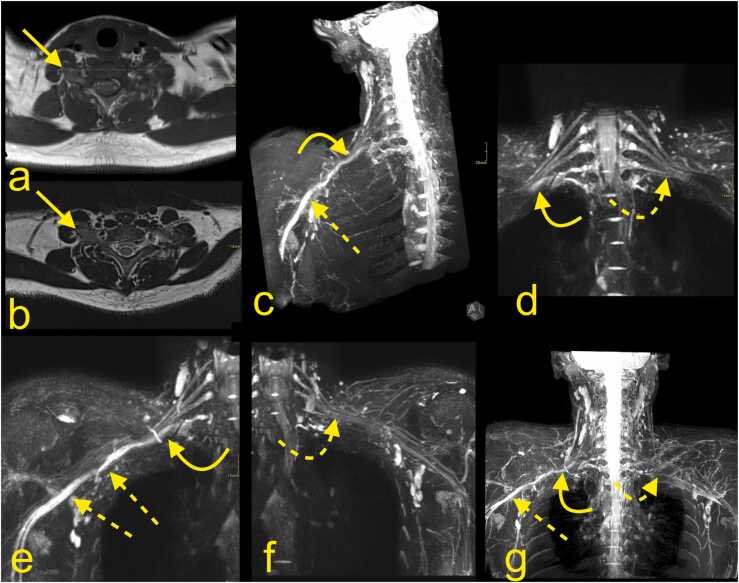
A 19-year-old patient presented with pain in the arm, forearm, and hand. A neurological examination showed a slight loss of strength in the intrinsic muscles and suspected thoracic outlet syndrome. Nerve conduction studies and EMG revealed the involvement of the lower trunk and medial cord. MRI of the BP with (a) T1-weighted TSE imaging, (b) T2 weighted TSE, and (c-g) MR neurography with (c, e, f, g) MIP reconstructions showed asymmetry of the BP (curved arrows) because of the presence of the cervical rib (straight arrow) on the right side, which is about 25 mm long. On the right side, the lower trunk (curved arrow) was displaced compared with the left side (curved dashed arrow). The medial cord and the median and ulnar nerves were swollen (straight dashed arrow).

The neurovascular bundle to the upper limb comes from the thoracic outlet and the neck. Anatomically, the thoracic outlet is limited by the Th1 posteriorly, the first rib laterally, and the manubrium sterni anteriorly. The thoracic outlet's central part contains structures passing between the neck and mediastinum, among other subclavian vessels, while the lateral part contains the apex of the lung covered by the pleura. The thoracic outlet space is related to other compartments like the scalene triangle containing the BP and subclavian artery, the costoclavicular space, and subcoracoid space with the entire neurovascular bundle [23], [37], [38]. The trunks and divisions of the BP and the subclavian artery and vein are superior to the pleura's cupula. Functionally and clinically, the space for structures passing through the thoracic outlet is limited by the insertions of the scalene muscles. Thoracic outlet syndrome (TOS) is caused by compression of the BP or the subclavian artery or vein (Fig. 8, Fig. 9). TOS may have a form of neurogenic or vascular disorder; thus, a patient may present with paresthesia, pain or muscle atrophy, limb swelling, and discoloration. The most common form is neurogenic TOS comprising 90% of TOS diagnoses [38], [39] (Fig. 15, Fig. 16). The diagnosis of TOS, especially neurogenic TOS, is predominantly clinical and based on the patients' symptoms and clinical findings. MRI plays an essential role in confirming the diagnosis and identifying possible morphological and pathological causes requiring surgery.

MRI findings, which can be suggestive for neurogenic TOS, are sometimes discreet. The most common, however non-specific, is asymmetry and edema in the BP (Fig. 15, Fig. 16). If dynamic MRI is performed, there is a loss of fat directly related to the BP in abduction, which may improve the specificity of the diagnosis [38], [39], [40]. Diagnostic imaging can show the cause for compression of the vessels or the BP and exclude other causes of the patient's symptoms. An accessory rib is present in about 6% of the population, but it is important to note that the presence of an accessory rib does not suggest TOS directly, especially in the absence of typical symptoms. Most cervical ribs are not connected to the first rib (Fig. 16). If they are, it may be a fibrotic or bony connection (Fig. 15). Rarely, other anomalies of the first rib or clavicle can be found to be the cause of TOS. Post-traumatic callus, scarring or deformation, exostosis, accessory fibrous bands, or accessory muscles may also compress the BP or influence the vessels [38], [40]. Performing bilateral examination with MRI and radiography is essential for the comparison of sides. It is essential to observe that dynamic changes in the vessel diameter might not be associated with the presence of vascular TOS, as the dynamic narrowing of vessels is often present in asymptomatic patients [50].

##### 1.5.4.4 Further prospects for BP magnetic resonance imaging

Water diffusion imaging of the brachial plexus may contribute to evaluating nerve tissue arrangements on the molecular level [8], [9], [41]. DTI allows the reconstruction of nerve fibers in 3D, assessing the water diffusion in different directions. The disorder of a nerve structure results in different diffusion values. Thus, DTI is a promising method that may help to differentiate between different brachial plexus pathologies [9], [41], [42], [43]. DTI allows visualization of long trajectories of the nerves and distinguishes them from the adjacent structures with a comparable signal on conventional MRI sequences [9]. Progression of nerve regeneration after surgery or trauma may be assessed using DTI [9]. However, usage of this method is challenging because artifacts from the inhomogeneity of BP orientation, head, and neck structures, and long acquisition time limit this method's use in clinical practice [8], [42], [44]. While DTI is a present an experimental method used mainly in research, there is a potential to develop the technology further and hope that it will become mainstream in the future.

However, with the progress of surgical treatment, this method will probably become more applicable in clinical practice [41], [42]. Three-dimensional (3D) constructive interference in steady-state (CISS) is a gradient-echo MRI sequence that may show root avulsion, focal pial adhesions, or traumatic syringohydromyelia better than conventional sequences [45]. 3D-imaging sequences may be instrumental in the assessment of the BP in the newborn.

##### 1.5.4.5 Summary

The identification and description of non-traumatic pathologies involving the brachial plexus need to be systematic and detailed based on anatomical localization because it includes diverse lesions. Imaging findings need to be correlated with clinical data to achieve a correct diagnosis. MRI may help narrow down the list of differential diagnoses and plan further invasive diagnostics such as biopsy or to plan radiotherapy.

## Ethics approval

Not applicable. No ethics approval is required for this educational review.

## Consent for publication

Not applicable. The manuscript does not contain the data of individuals in any form.

## Funding

This project received no funding.

## Authors' contributions

PS conceived the idea of the pictorial review to publish in this study. PS selected appropriate MRI figures. PS, MG, BC, AMG analyzed and annotated the chosen images. BC prepared anatomical specimens. PS, AMG, and MG wrote the first draft of the manuscript. All authors read and approved the final manuscript.

## Declaration of Competing Interest

The authors declare that they have no known competing financial interests or personal relationships that could have appeared to influence the work reported in this paper.

## Data Availability

Yes.
